# Supplementary material for: A single donor is sufficient to produce a highly functional in vitro antibody library
Source: Commun Biol. 2021 Mar 19;4:350. doi: 10.1038/s42003-021-01881-0 (PMC7979914; doi:10.1038/s42003-021-01881-0)
Supplement: Supplementary file 2 — Supplementary Information [file 42003_2021_1881_MOESM2_ESM.pdf]

Supplementary information.

Supplementary table 1. Chain productivity.

Status	Total Heavy MiSeq	Total Light Chain MiSeq
Productive & Unproductive	12,171,283	743,129
Productive	10,785,580	624,291
Productive Reads	89%	84%

Tabulated number of productive and unproductive heavy chain sequences from MiSeq; 89% of sequences are productive (no frame-shifts, stop codons or low-quality base-pair called). Tabulated number of productive and unproductive light chain sequences from MiSeq. 84% sequences are productive.

Supplementary table 2. Relative primer promiscuity for each IGHV primer set.

	Gene On-target	Gene Off-target	Family On-Target	Family Off-Target
**IGHV1a**	60	40	62	39
**IGHV1b**	0.2	99.8	99.4	0.6
**IGHV1c**	60	40	85	15
**IGHV1d**	6	94	67	33
**IGHV2a**	77	23	96	4
**IGHV2b**	61	39	99	1
**IGHV3a**	30	70	53	47
**IGHV3b**	22	78	60	40
**IGHV3c**	45	55	70	30
**IGHV4**	96	4	98	2
**IGHV5a**	31	69	31	69
**IGHV6a**	50	50	50	50

Supplementary table 3. Clonotype analysis.

	GM-CSF	OX40	B7-H4	CD40-L
**Unique HCD3_Clonotypes Hamming ≤ 1, ≥ 2 Reads**	386	234	395	394
**Unique HCD3_Germline Clonotypes Hamming ≤ 1, ≥ 2 Reads**	905	452	1137	913
**# Reads**	62689	59780	55013	54881

Number of the different HCDR3 clonotypes identified after NGS analysis of the selection campaign outputs. HCDR3 are clustered into a "Unique HCDR3 clonotype) if the Hamming distance is ≤1 and abundance > 1. Clones are considered to belong to an "Unique HCDR3-Germline clonotypes" if the Hamming distance is ≤1, they belong to a different IGHV germline and the abundance is > 1).

Supplementary Figure 1.

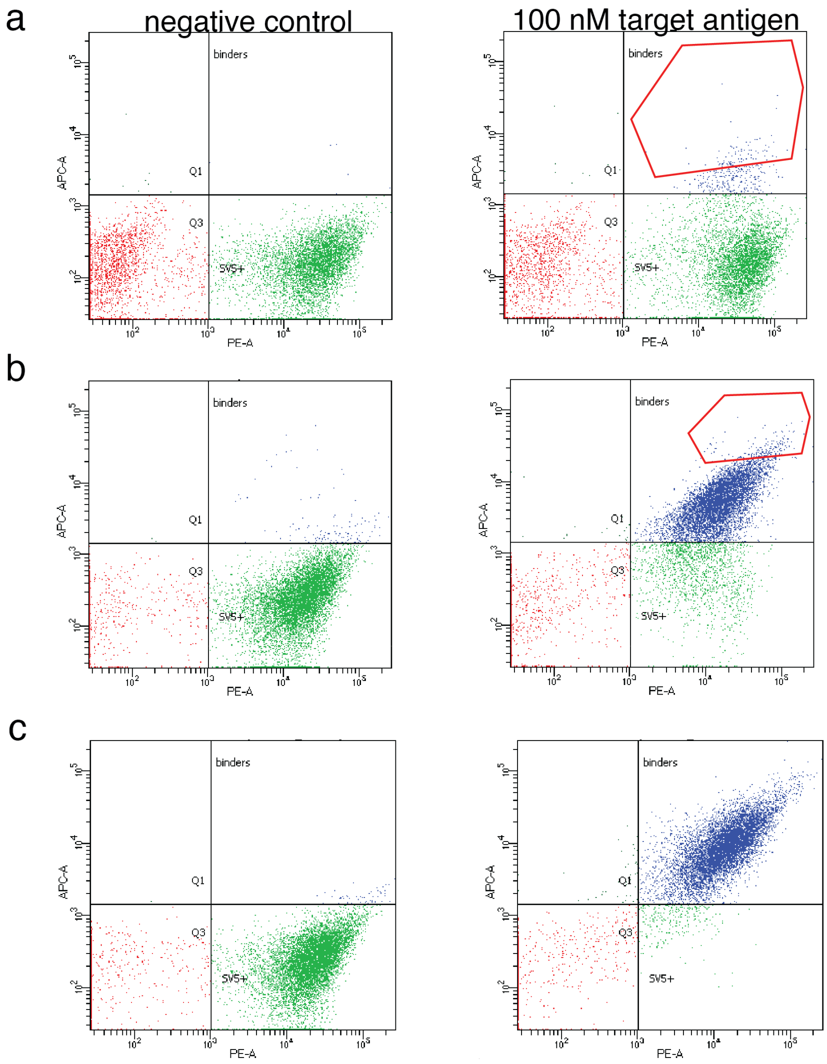

The yeast cells were analyzed simultaneously for: i) the phycoerythrin signal (PE), related with the display of the scFvs thanks to the detection of their V5 tag with and anti-V5 PE-conjugated antibody, ii) and allophycocyanin signal (APC) related with the detection of the biotinylated antigen by the scFvs thanks to the use of strepatavidin-APC conjugated. Four gates were generated. The “binders” gate represents double positive cells: scFvs are displayed and they bind to the target. In this gate the sort gate was established (in red). **a** Right after the subcloning of the phage selection output into yeast a sorting gate is designed corresponding to ~0.5-1% of the scFv-displaying yeast cells. **b** During the analysis of the cells after the first sort, a sorting gate is designed corresponding to ~0.5-1% of the scFv-displaying yeast cells. **c** The resulting population after two rounds of sorting show that all the displaying yeast cells bind to the specific antigen and not the negative control one.
