# Supplementary material for: A single donor is sufficient to produce a highly functional in vitro antibody library
Source: Commun Biol. 2021 Mar 19;4:350. doi: 10.1038/s42003-021-01881-0 (PMC7979914; doi:10.1038/s42003-021-01881-0)
Supplement: Supplementary file 3 — Reporting Summary [file 42003_2021_1881_MOESM3_ESM.pdf]

nature research

Corresponding author(s): Andrew Bradbury

Last updated by author(s): 02/01/2021

Reporting Summary

Nature Research wishes to improve the reproducibility of the work that we publish. This form provides structure for consistency and transparency in reporting. For further information on Nature Research policies, see our Editorial Policies and the Editorial Policy Checklist.

Statistics

For all statistical analyses, confirm that the following items are present in the figure legend, table legend, main text, or Methods section.

n/a	Confirmed	
☐	☒	The exact sample size (*n*) for each experimental group/condition, given as a discrete number and unit of measurement
☐	☒	A statement on whether measurements were taken from distinct samples or whether the same sample was measured repeatedly
☐	☒	The statistical test(s) used AND whether they are one- or two-sided<br>*Only common tests should be described solely by name; describe more complex techniques in the Methods section.*
☐	☒	A description of all covariates tested
☐	☒	A description of any assumptions or corrections, such as tests of normality and adjustment for multiple comparisons
☐	☒	A full description of the statistical parameters including central tendency (e.g. means) or other basic estimates (e.g. regression coefficient) AND variation (e.g. standard deviation) or associated estimates of uncertainty (e.g. confidence intervals)
☐	☒	For null hypothesis testing, the test statistic (e.g. *F*, *t*, *r*) with confidence intervals, effect sizes, degrees of freedom and *P* value noted<br>*Give P values as exact values whenever suitable.*
☐	☒	For Bayesian analysis, information on the choice of priors and Markov chain Monte Carlo settings
☒	☐	For hierarchical and complex designs, identification of the appropriate level for tests and full reporting of outcomes
☒	☐	Estimates of effect sizes (e.g. Cohen's *d*, Pearson's *r*), indicating how they were calculated

Our web collection on statistics for biologists contains articles on many of the points above.

Software and code

Policy information about availability of computer code

Data collection	The sequencing data were obtained as fastaq files.
Data analysis	Open source codes were used for the data analysis: R and within R biostrings, IgBlast, peptides, stringdist. All the specific codes used are listed in the manuscript.

For manuscripts utilizing custom algorithms or software that are central to the research but not yet described in published literature, software must be made available to editors and reviewers. We strongly encourage code deposition in a community repository (e.g. GitHub). See the Nature Research guidelines for submitting code & software for further information.

Data

Policy information about availability of data

All manuscripts must include a data availability statement. This statement should provide the following information, where applicable:

- Accession codes, unique identifiers, or web links for publicly available datasets
- A list of figures that have associated raw data
- A description of any restrictions on data availability

The data that support the findings of this study are available from the corresponding author upon reasonable request.

Field-specific reporting

Life sciences study design

All studies must disclose on these points even when the disclosure is negative.

Sample size	N/A the manuscript analyze an antibody library obtained from a single individual
Data exclusions	No data were excluded from the analysis
Replication	The deep antibody library analysis was performed once and was able to generate billions of antibody reads. The codes used in the manuscript were designed to account and evaluate the quality of the analysis.
Randomization	N/A
Blinding	N/A

Reporting for specific materials, systems and methods

We require information from authors about some types of materials, experimental systems and methods used in many studies. Here, indicate whether each material, system or method listed is relevant to your study. If you are not sure if a list item applies to your research, read the appropriate section before selecting a response.

Materials & experimental systems

n/a	Involved in the study	
☐	☒	Antibodies
☐	☒	Eukaryotic cell lines
☒	☐	Palaeontology and archaeology
☒	☐	Animals and other organisms
☒	☐	Human research participants
☒	☐	Clinical data
☒	☐	Dual use research of concern

Methods

n/a	Involved in the study	
☒	☐	ChIP-seq
☐	☒	Flow cytometry
☒	☐	MRI-based neuroimaging

Antibodies

Antibodies used	V5 Tag Monoclonal Antibody (2F11F7) ThermoFisher #37-7500 conjugated in house with R-Phycoerythrin PE / R-Phycoerythrin Conjugation Kit - Lightning-Link® (ab102918)
Validation	The V5 Tag Monoclonal Antibody conjugated with PE has been widely used by our group and other. Here are some of our citation were the antibody has been previously validate:<br>- Ferrara, F., et al., Using phage and yeast display to select hundreds of monoclonal antibodies: application to antigen 85, a tuberculosis biomarker. PLoS One, 2012. 7(11): p. e49535.<br>- Ferrara, F., et al., Recombinant renewable polyclonal antibodies. MAbs, 2015. 7(1): p.32-41.

Eukaryotic cell lines

Policy information about cell lines

Cell line source(s)	Saccharomyces cerevisiae Meyen ex E.C. Hansen (ATCC® MYA-4941™) strain EBY100<br>The expression of the IgGs were performed by a CRO using HEK293 cell line.
Authentication	Cells were commercially available and authenticated by the distributor (ThermoFisher for the HEK293; ATCC for S.cerevisiae)
Mycoplasma contamination	Cells were always negative for mycoplasma contamination
Commonly misidentified lines<br>(See ICLAC register)	N/A

Flow Cytometry

Plots

Confirm that:

- [x] The axis labels state the marker and fluorochrome used (e.g. CD4-FITC).
- [ ] The axis scales are clearly visible. Include numbers along axes only for bottom left plot of group (a 'group' is an analysis of identical markers).
- [ ] All plots are contour plots with outliers or pseudocolor plots.
- [ ] A numerical value for number of cells or percentage (with statistics) is provided.

Methodology

Sample preparation	Yeast cells were washed by adding washing buffer (PBS, 0.5% BSA and 50 nM EDTA), spin down and buffer was removed. Cells were incubated 30 min with the antigen solution, washed again, incubated with the anti-V5 antibody and streptavidin-Alexa-Fluor-633, washed again and analyzed.
Instrument	BD FASCAria III
Software	BD Diva
Cell population abundance	N/A
Gating strategy	Double positive yeast cells were sorted: positive for expression (PE signal on the X-axis) and display of the scFVs on their surface detectable with V5-PE antibody, and positive for the binding of the biotinylated antigen by the scFvs detected using fluorescently labeled streptavidin (APC signal on the Y-axis).

- [x] Tick this box to confirm that a figure exemplifying the gating strategy is provided in the Supplementary Information.
